# Book Reviews

**Published:** 1820-07

**Authors:** 


### CRITICAL ANALYSIS

OF

### RECENT PUBLICATIONS, IN THE DIFFERENT BRANCHES OF MEDICINE AND SURGERY;

### SELECT MEMOIRS, AND HISTORIES OF CASES;

### In the Literature of Foreign Nations.

Πατρίδες ἄρα
Ανδράσιν, ὦ πατραῖς ἄνδρες, ἀγαλλόμεθα.

---

*Medico-Chirurgical Annuary of the Civil Hospitals and Infirmaries of Paris; or, a Collection of Memoirs and Observations by the Physicians and Surgeons of those Establishments.* 4to. pp. 636; with fourteen Plates. Paris, 1819.

[In continuation from vol. xliii. p. 517.]

THE memoir next in order in this work is by M. MONGENOT, and relates to

*The Principal Facts observed at the Hospital for Sick Children, during the Year* 1815.

It commenced with a summary of general observations relating to "fifty-six cases of ophthalmia which reigned epidemically." These cases occurred towards the middle of December 1814, in the division of the hospital appropriated to boys, and chiefly in a ward facing east and west in its longitudinal direction, and having its exposed extremity open to the north, the southern end being in contact with the general building. The season was cold and moist. The ophthalmia suddenly attacked five or six children, and in a few days afterwards the number of patients amounted to eighteen or twenty. The children affected were, for the most part, weak, puny, scrofulous, or convalescent from other diseases.

The author distinguishes three periods in the course of the disease. At the commencement, the patients suffered head-ach, and a pruritus about the eyes that obliged them to rub the parts; and there was an abundant flow of tears. There soon afterwards appeared redness, and injection of the conjunctiva and margin of the eye-lids, which became tumefied; the impression of light was painful; and from this time there was a discharge of pellucid mucus.

During the second period, which began on the third day, the conjunctiva was very red and much tumefied throughout its whole extent, except over the cornea, which appeared sunk one or two lines below the rest of the surface of the conjunctiva. The eyelids, the upper especially, were swelled, and of a deep-red colour. There was a copious discharge of white, puriform, matter; and ordinarily the margin of the lower eye-lid was hidden behind that of the upper. When an attempt was made to open the lids, a large quantity of purulent matter ecaped; and, when they were sufficiently separated, the conjunctiva appeared to be as it were detached from the surface beneath it: it was puffed up, and presented a granulated appearance like the inner surface of the stomach. A few days afterwards, the upper lid became of a violet colour; the matter which flowed from the eye was yellowish, and had a bloody appearance. The conjunctiva was of a less bright red hue, and it appeared soft, lax, and tumefied.

The whole of the foregoing symptoms continued for about twelve or fifteen days, when, at the end of this time, or sometimes earlier, the tumefaction and redness diminished, and then the third period of the disease commenced. The purulent matter was now less abundant, and lost its opacity; the patients could open the eye-lids, and the symptoms gradually disappeared.

In a few cases the disease was rebellious to all the remedial measures that were employed. One patient lost both eyes; another had one eye totally destroyed; and three lost the sight of one eye: one of the latter, who was previously blind in one eye, lost the good one. Fifty were perfectly cured. The duration of the epidemy, by the succession of patients, was somewhat more than two months.

M. Mongenot does not appear to have examined whether or not the affection was contagious as well as infectious. Some observations on this point are desirable, as well as an account of the age of the patients in general. It would be interesting, also, to know whether or not they appeared to suffer disease of the mucous membranes of other parts of the body at the same time.

The *treatment* consisted in the application of leeches at the onset of the disease, and, in a great proportion of cases, they were applied to the legs; in a few, a vein was opened in the foot. In all of them, emollient and sedative collyria were used, with irritating pediluvia and embrocations to the feet. Vesicatories, or a seton in the neck, according to the severity of the disease, and emetics, were also employed. Whey, made a little sharp, constantly produced more benefit than emetics given to excite vomiting. The topical application which produced most ease and amelioration of the affection, was a cataplasm of the pulp of apples, with the addition of opium and acetate of lead.

Histories of eight cases of croup, and seven of peritonitis, and twenty-four of mucous fevers, follow, which contain nothing novel of importance. A case of chorea is detailed; the subject of which was a boy six years of age, and whose mother had the same malady in her youth. It came on without any evident cause. The treatment consisted in a warm bath daily, pills composed of camphor, nitre, musk, castor, and extract of valerian. After a few days, a cupful of decoction of cinchona and valerian, three times a-day, was added to the remedies previously mentioned. The termination was favourable. M. Mongenot states, that he has always found the same means successful in similar cases. When the patient is of a strong constitution, he has ordinarily directed blood-letting from the arm and an emetic, as precursory measures.

The next paper is on

*The Inflammation of the Globe of the Eye, which reigned epidemically, in* 1818, *at the Hospital for Sick Children.* By M. Jadelot.

The prevalent diseases had been, for a long time, inflammatory; hardly any cases of ophthalmia had been seen, when, during the summer, affections of this kind began to appear in a great number of children. Those who were attacked with measles or small-pox were, in the first instance, most disposed to contract the ophthalmia.

When the epidemy first appeared, the affection assumed an acute character, but it was not very severe in degree: it was confined to an inflammatory tumefaction of the conjunctiva lining the eyelids; and in several cases passed into a chronic state, in weak or scrofulous subjects, being at the same time often reduced to a slight redness of the margin of the eyelids, accompanied with a thick mucous discharge.

In the acute stage, purgatives were not successful; the mildest produced more or less severe inflammation of the mucous membrane of the stomach and intestines, inflammation of which was, at the same season, the most common disease. The application of leeches to the temples, emollient collyria; and, when the redness of the conjunctiva persisted though the pain was diminished, sedative collyria; and, when the pain continued after the redness had disappeared, opiate collyria; were the remedies employed. They were generally successful; and in the cases where old, and as it were constitutional, cases of ophthalmia had suddenly assumed an acute character, they were ordinarily sufficient to reduce the disease to its previous state.

In the chronic stage, amongst the revulsive discharges, experience showed that setons in the neck were preferable to blisters.

Ophthalmia continued to prevail during the autumn, and it was then that the affection acquired a very severe degree of intensity. The inflammation was no longer confined to the external parts, but attacked especially the "globe of the eye." These cases also, as well as those which occurred during the preceding season, appeared first in some children during the progress, or towards the end, of small-pox; then in those who were affected with other acute cutaneous phlegmasiæ, or with herpetic eruptions in the face, prurigo, or chronic ophthalma; but the epidemy in its progress did not spare children of any age, of good constitutions, and who had previously been in perfect health.

"The malady almost always appeared after a fit of shivering, either from the children having been exposed for some time to a current of cold air, or to the cold and humid atmosphere of the evening; sometimes it seemed to be occasioned by too severe and prolonged impression of the light of the sun, or by a contusion on the temple."

Both eyes ordinarily participated in the inflammation, but successively, so that it did not become very severe in the one last affected until it had begun to subside in the other.

From the commencement, the patients could not bear the light, the pupils were contracted, and vision was almost impossible. There were present severe head-ach, intense fever, a full and hard pulse, ardent heat of the skin, and delirium. The eyelids soon became red, engorged with blood, tumid, and more or less distended by the tears which were collected in great abundance between them and the globe of the eye. The conjunctiva of the eye, when it could be inspected, was more or less tumid and injected with blood. The cornea was turbid; and it sometimes presented a whitish appearance, which began at its inferior part, and soon spread throughout its whole extent; but it was always difficult, and sometimes impossible, at this stage of the disease, to separate the eyelids sufficiently to permit the eye to be viewed.

The lancinating pains which were felt in the bottom of the orbit from the invasion of the malady, soon became intolerable, as well as the head-ach; and, in some instances, produced a permanent contraction of the muscles of the face of the corresponding side. The thirst was ardent, the tongue red; the stools and urine but rarely evacuated; and in the evening and during the night, an exacerbation of the fever took place, attended with great agitation and violent delirium.

As the disease proceeded, the cornea became more and more protuberant and unequal in its surface; abscesses of considerable size sometimes formed in the substance of the eye-lids; in other cases, an abundance of thick, puriform, matter issued from between them.

From this time, if efficacious treatment was not resorted to, the loss of sight was the least danger the patients encountered. The inflammation of the eye increased so rapidly, that the loss of one or two days became irreparable. The peril was imminent when this affection was complicated with small-pox, or with some abdominal inflammation; or when it coincided with the first dentition, especially in weak infants. In the latter circumstances, affections of the brain, and even hydrocephalus, were the consequences.

Experience soon showed that all the means ordinarily used in the treatment of severe ophthalmia, were inefficacious in this affection. The different revulsives applied to the surface of the body or administered inwardly, emollient collyria, acidulated drinks, the application of leeches to the temples, and even blood-letting by the lancet, in the foot or arm, did not apparently diminish the severity of the symptoms; whilst "the only efficacious remedy was not resorted to:" this was bleeding from the jugular vein. It was necessary to employ it at the commencement, and to repeat it several times in a few days. "Used in time, and to a great extent, in proportion to the age and constitution of the child, it had constantly the most happy success: it suppressed the disease suddenly, if that were still possible; or, at least, in all cases, it produced all the alleviation that could be expected at the period at which the inflammation had arrived. The delirium and head-ach ceased, the fever diminished, the local symptoms lost their violence; the eyelids, become less painful, might be separated; and, when the eye was not so much disorganized as to render the re-establishment of sight impossible, it was remarked, that the pupils admitted the rays of light without much uneasiness."

The cure being thus commenced, the other means of treatment were employed with advantage: thus, it was necessary, in some cases, to apply leeches to the neighbourhood of the eye several times, in order to destroy completely the inflammation. But, M. Jadelot says, "purgatives were never proper; they excited too much the intestines, without ameliorating the state of the eyes." "The use of astringent collyria was almost entirely abstained from, because, in the decline of the disease, they readily renewed the local irritation. Emollient collyria, continued until the termination, appeared necessary to prevent the return of this irritation." There never was occasion, M. Jadelot states, "to have recourse to puncture of the globe of the eye, nor to excision of the cornea. When the globe was in suppuration, the detachment of the cornea and its partial destruction, which promptly took place, procured an easy and sufficient evacuation for the pus."

This form of the epidemy, which commenced in autumn, continued to exist during the ensuing winter; but the cases were then less numerous and less severe.

The state of the eyes was carefully examined in those who died either during the existence of the disease, or soon after its termination. "The cornea was found softened, infiltrated, thickened, detached at its circumference, and partially separated from the sclerotica; abscesses were found between its laminæ, ulcers on its anterior surface, and there was pus accumulated at its posterior surface, or adhesions of it with the iris and the crystalline. The crystalline itself was opaque and tumid; the vitreous humour turbid and traversed by red vessels, or replaced by a substance similar to black blood, partly coagulated; and, lastly, the globe of the eye was more or less destroyed by suppuration."

Several cases are related by the author, which illustrate the foregoing observations, and comprise some other more or less interesting particulars; and they prove most fully the propriety of his remarks on the importance of blood-letting from the jugular vein, and the utility of the application of leeches after that measure had been resorted to. A seton in the neck appeared to be very beneficial, when the inflammation had been somewhat relieved. One patient, a boy sixteen years of age, remarked the phenomenon of *muscæ volitantes;* and another, of twelve years, said that every thing seemed red to him. Both these cases terminated favourably. In the latter, the jugular vein was opened twice in one day, and once on the following day; after which, leeches were repeatedly applied to the temples. The quantity of blood taken from a patient of ten or twelve years of age, each time the vein was opened, appears to have been about six ounces. There is but little to be desired in the account given of this epidemy by M. Jadelot; but the history would have been more complete, had he related more particularly the ordinary consequences of the disease in its less favourable modes of termination, when the patients survived.

The next paper comprises some *Observations on different Cases of Parturition*, by Madame LACHAPELLE, sage-femme in chief of the Maison d'Accouchement.

There is not one of these cases that presents any thing of importance, in regard either to rarity or mode of treatment; but we must pay a passing compliment to Madame Lachapelle for her acquirements in anatomy, (which she is, however, rather fond of displaying,) and her judicious practical conduct. Though we pass over her memoir without appropriating any part of it to our Journal, we are pleased to see it in the volume before us, as it exemplifies the propriety of that part of her national police which has placed her at the head of an hospital; and it is gratifying to reflect on the liberal sentiments that caused her production to appear amongst those of several of the most eminent physicians of the French capital.

The next memoir is entitled *Observations collected at the Maison de Santé of the Faubourg Saint-Martin;* by MM. DUBOIS, surgeon-in-chief, and GUERBOIS, assistant-surgeon, of that establishment.

The three first cases are devoid of remarkable interest; of the fourth we shall give an abstract. A man, forty-two years of age, of a robust constitution and sanguine temperament, was sent from one of the provinces of France, that he might suffer the operation of amputation of his penis; and the patient himself desired that it might immediately be effected, believing that it was the only means by which his life could be preserved; for it was supposed that the part in question was affected with cancer. It had acquired a prodigious size; the prepuce was much tumefied; the glans was of a reddish-brown colour, very hard, and presented at its anterior surface a fungous tumor ("*champignon*"), of about an inch and a half in diameter in every direction: this tumor was situate on the corona glandis and internal surface of the prepuce. The patient suffered a good deal of pain. There was no enlargement of the inguinal glands.

A course of laxative medicines and mild narcotics was ordered for him; and he was directed to bathe the penis with a mucilaginous and opiate lotion for three or four hours daily. On the eighth day after his entry into the Maison de Santé, the fungous tumor was removed by M. Dubois. He made two elliptic incisions round its basis, and then, raising it with pincers, detached it by one stroke of the bistoury. Hardly any hemorrhage ensued. The dressing was simple, and was removed on the third day. At this time, the colour of the glans was more healthy, and this part was less hard; the tumefaction of the prepuce had somewhat subsided; the pain had almost wholly ceased. The symptoms subsided from day to day, and the patient returned home at the end of five weeks, perfectly cured.

Nothing is said respecting the previous history of this affection, except that, after stating the query, "What was the nature of this disease?" it is remarked by M. Guerbois, that "it appears that the use of mercurials internally and externally, and of irritating applications to the champignon, had produced the engorgement and the series of accidents which were successively developed."

The other cases related in this paper are not sufficiently remarkable, to induce us to occupy the pages of our Journal with an account of them. We must however mention, that they do credit to the practical talents of the writers.

M. Pinel has contributed to this production some *General Remarks on the Medical Constitution of the Decline of the Summer and of the Autumn of the Year* 1815, *in relation to Observations made at the Hospital de la Salpetrière.*

This hospital is inhabited by females only, who are either aged or infirm, or affected with mental derangement. It is to the former class of persons that the observations of M. Pinel expressly relate. During the great and continual heat of the summer, affections of the stomach and intestines were the prevalent forms of disease; appearing sometimes with the symptoms of total loss of appetite, disgust for food, a bitter taste in the mouth, dull pain in the region of the stomach, &c. In other cases, there was more or less acute pain, with a discharge from the bowels of yellowish serous fluids, which only required acidulated mucilaginous drinks, mixed with a little wine, according to the strength of the patient, and which terminated favourably under the use of those means. Sometimes gastric fevers, of a more or less regular type, and marked by paroxysms attended with a sense of burning heat, but without danger, appeared, and which terminated equally favourably by the use of the drinks above mentioned.

An hospital inhabited by several thousand persons, almost all of a very advanced age, submitted to a uniform mode of life during the whole of the year, constantly dressed and fed in the same manner, always exposed to depressing moral affections, and confined to a sedentary life, could not fail to present numerous instances (M. Pinel remarks), of obstinate catarrhal affections of the chest towards the decline of the autumn, which in this year was unusually cold. So the gastric disorders were replaced, in November and December, by pulmonary catarrhs of various characters, and peripneumony and pleurisy, either simple or complicated with typhous fever. The catarrh assumed an acute form, with febrile symptoms; the cough and expectoration arrived at their greatest degree of intensity in a few days, and then gradually subsided and disappeared under the use of merely some acidulated and edulcorated drink. In a few cases they became chronic, and terminated in phthisis.

The gradual progress of typhous fever might be daily predicted by the appearances of the tongue. At first, it was brownish, then black and dry; then had a fuliginous aspect for a few days; lastly, by a retrograde course, it became at first moist at the edges, then deprived by degrees of its black and glutinous coat, and this was attended with a gradual return of all the vital functions to the natural state.

The cases of typhous fever accompanied with peripneumony were almost always mortal: none of the means resorted to for their relief seemed to be of apparent advantage. Two or three days had hardly elapsed, when the expectoration gradually diminished, and at length was suppressed. The oppression of the chest increased; the sense of anxiety became extreme; and the patient died apparently by a sort of suffocation, from the impossibility of inspiration.

*Some Chirurgical Observations.* By M. Lallemand, Professor of the Faculty of Medicine, Surgeon-in-chief of la Salpetriere, &c.

The first case here related is sufficiently rare and interesting to induce us to give M. Lallemand's account of it in detail.

"Marguerite Recorda, twenty-three years of age, of a plethoric temperament, requested my assistance for a slight ophthalmia. She had been from her infancy in a state of imbecility, and had a tumor on the occiput, which, at first of the size of a nut, had at length acquired that of a hen's egg. This tumor, mobile, indolent, of a moderately-firm consistence, with a narrow base, presented all the characters of an encysted tumor.

"Having determined on the operation, I circumscribed by an incision the base of the tumor, and then proceeded to its dissection. But I was soon struck by a clear, white, and shining appearance, which I observed at one part of its base: this appearance was also developed in several other points, and I thought I recognized in it the aspect of the dura mater. I communicated my doubts to the pupils present, and, by passing my fore-finger into the incision, I discovered that the base of the tumor was surrounded by a bony circle formed by the substance of the os occipitis. I suspended the operation, and declared to the pupils that I feared a fatal result. The wound was dressed, and the patient placed in bed: she suffered no inconvenience during the day of the operation. On the ensuing day, there came on violent head-ach, with a hard pulse, (*blood-letting from the arm, laxative drinks*,) prostration of strength, vomiting of greenish bile occurring more and more frequently, rebellious to antispasmodics, &c. and continuing until death, which happened on the eighth day after the operation.

"*Dissection.*—On the vault of the cranium being raised, the cerebrum appeared in the natural state; but the portion of the dura mater which forms the posterior part of the tentorium of the cerebellum was engaged in an opening, of a regular circular figure, three lines in diameter, formed in the occipital bone. This production of the dura mater was covered on its external surface by very dense cellular tissue. Its internal surface enclosed a prolongation of the two lobes of the cerebellum, of the size of a nut; and several co[illegible]ctions of purulent matter were discovered in the substance of this organ."

Some days after the communication of the account of this case to the Faculty of Paris, M. Buffos, surgeon-in-chief to the Hospital for Children, requested M. Lallemand to see one of his patients, whom he suspected to have a similar affection. M. L. confirmed his suspicion; and the truth of his opinion was soon after proved, on the death of the patient. "The body, on being opened," says M. L. "presented a tumor situate in the same place, and formed of the same parts, as those which I described in the case above mentioned." This is the whole of the account furnished of this second case.

M. Lallemand also briefly relates an account of the appearances on dissection of a case of hernia of the uterus. "This organ," he says, "with all its dependencies, escaped from the cavity of the belly by the crural arcade, and descended as far as the middle of the right thigh. The vagina was so much elongated, that its insertion into the uterus did not commence until it passed to the distance of three fingers' breadth below the fold of the groin."

M. Lallemand apologizes for the imperfect manner in which the foregoing cases are described, by saying that an attack of sickness had prevented him from relating them in a manner consonant with his wishes.

*Observations on some Diseases of the Cerebellum, the Cerebrum, and their Membranes; collected at the Hospital de Bicetre.* By M. Hebreart, Physician in Ordinary to the Insane Patients in that Hospital, &c.

We shall give the cases of disease of the cerebellum here related in detail, as they present many interesting circumstances, especially in the great prostration of strength, and as it were gradual extinction of the functions of the body from want of cerebral nervous influence, which accompanied the progress of the disease of the cerebellum; and, in the third case, the state of respiration for several days before death, is equally remarkable. When we consider the origin of the eighth pair of nerves, and their influence on the functions of the organs here apparently particularly affected, we shall not find it very difficult to understand the relations of these phenomena.

"A young man, who had been for six years in a state of idiotism, occurring consequent to excessive study, was attacked, during the winter, with a scorbutic affection, to which he fell a victim, after having presented, during the last month of his life, not only the debility which is common in scorbutic affections, but also that state of prostration, with dryness of the tongue and lips, which characterizes adynamic fevers. On opening the body, the four ventricles were found distended by a very large quantity of serous fluid, and what was more worthy of remark, was a peculiar degeneration of the parietes of the fourth ventricle, which were of a yellowish colour and of a lardaceous consistence: this degeneration extended more than the space of a line into the substance of the cerebellum."

"A man of a robust habit arrived at the age of fifty years in following his labour as a stone-cutter. Assuredly this avocation is not calculated to excite the passions: however, he suffered, in 1811, an access of mania, which continued for three successive months without lucid intervals. Nevertheless, either by time, or by the debilitating means to which he was submitted at the Bicêtre, the patient having recovered his reason, was claimed by his wife, when he had resided there for six months, and resumed his labour, which he continued for about two years; but it was found necessary to bring him to the hospital again in 1813. He did not on this occasion experience any exaltation of his faculties, but he was in such a state of physical and mental inertia, that he had neither the strength nor intelligence necessary to direct his saw: yet the bulk of his limbs was not diminished, and the functions of the body appeared to be freely performed. His speech was indistinct, as much from the absence of ideas as from the commencing paralysis of the muscles of the tongue. Memory was entirely obliterated. This state lasted for several months; but insensibly passed into a sort of adynamic fever, characterized by dryness of the tongue and teeth, and general prostration of strength, which was not removed by the administration of cinchona, etherial and camphorated mixtures, mustard pediluvia, and the application of vesicatories to the legs. Still the pulse and the heat of the body differed but little from the natural state; and no re-action was observed, as occurs in true adynamic fever. On this state being thus prolonged, some light nutritious food was given to the patient: he digested it, but did not suffer any change either for the better or worse. His debility, however, insensibly increased, and he ceased to exist, after having passed fifty-five days without speaking or moving in his bed, and without having suffered any nervous commotion, nor any symptom of irritation. He did not even seem to feel the gangrenous eschars which were formed from lying so long in one position, about the sacral region and the trochanters of the femures. On the dissection of the body, the viscera of the thorax and abdomen presented nothing remarkable; but all the parts in the cranium were infiltrated with blackish blood. The lateral ventricles were dilated by a large quantity of serous fluid. But, what most elicited attention, was a gelatinous disorganization in the left lobe of the cerebellum. The whole of the inferior surface of this lobe was of the consistence of jelly, to the extent of three or four lines in its substance. It was only necessary to scrape gently with the handle of the scalpel the parts thus altered, to reduce them into a fluid state. This part of the cerebellum, which is convex in the natural state, presented then a large concavity, the parietes of which, hard and smooth, formed a sort of cyst, terminated inferiorly by the pia mater and the arachnoid membrane, which appeared to be altered in their organization without being destroyed."

M. Hebreart thinks, with apparent propriety, that the access of mania which the patient suffered in 1811, was owing to inflammation, which had, undoubtedly he says, preceded the alteration of the cerebellum, and that this inflammation, having become chronic, had given rise to the disorganization above described.

"An insane subject, who had been considered incurable for several years, because he suffered paroxysms of mania after irregular intervals, was attacked with an accidental malady, which appeared to be, at first, only a gastric affection, but which soon presented well-marked adynamic symptoms, such as prostration of strength, red and dry tongue, crusted teeth, &c. This state continued for nearly four months, in spite of the administration of tonics and stimulants, which produced no amelioration. No crisis took place; and the patient died, after having had the *rattles* for eight days. On opening the body, there was found at the inferior part of the cerebellum, of the right side, a disorganization, about six lines in diameter, of the substance of that organ, which had become hard, lardaceous, and of a yellowish colour; and this occupied not only the cortical part, but extended irregularly to the depth of several lines in the medullary substance. The pia mater was destroyed at the spot which corresponded with this alteration, and the dura mater had assumed the yellow colour of the diseased portion of the cerebellum."

M. Hebreart then relates some cases of disease of the cerebrum, with the view of showing how much alteration of the structure of this organ may exist, without any permanent sensible derangement of the faculties commonly attributed to it. But examples of a similar kind are too common to permit us to think those above alluded to proper for transcription. He also adduces some cases where the patients had died apparently from phrenitis, and yet the contents of the cranium showed no morbid appearances on dissection. These cases are less obscure than those of the foregoing kind, because it is easy to conceive that functional may exist without sensible organic lesion, or that various states of organic lesion may exist that are not perceptible to the senses; and, from a work recently published on

Mental Alienation, by the younger Pinel, it would appear that this affection more frequently depends on disease of some of the thoracic and abdominal viscera, than on a primary affection of the brain. In one of the cases related by M. Hebreart, there was, however, no apparent disease in any part of the body of a man who died on the seventeenth day from the accession of phrenitis. The only remarkable appearances were, that the heart was very firm, and the cerebral substance, especially that of the spinal marrow, had more consistence than ordinary.

